# Plasma derived from human umbilical cord blood: Potential cell‐additive or cell‐substitute therapeutic for neurodegenerative diseases

**DOI:** 10.1111/jcmm.13898

**Published:** 2018-10-18

**Authors:** Jared Ehrhart, Paul R. Sanberg, Svitlana Garbuzova‐Davis

**Affiliations:** ^1^ Saneron CCEL Therapeutics, Inc. Tampa Florida; ^2^ Center of Excellence for Aging and Brain Repair Morsani College of Medicine University of South Florida Tampa Florida; ^3^ Department of Neurosurgery and Brain Repair Morsani College of Medicine University of South Florida Tampa Florida; ^4^ Department of Pathology and Cell Biology Morsani College of Medicine University of South Florida Tampa Florida; ^5^ Department of Psychiatry Morsani College of Medicine University of South Florida Tampa Florida; ^6^ Department of Molecular Pharmacology and Physiology Morsani College of Medicine University of South Florida Tampa Florida

**Keywords:** cell apoptosis, cell viability, cord blood plasma, cytokines, growth factors, human umbilical cord blood, mononuclear cells

## Abstract

Limited efficacy of current therapeutic approaches for neurodegenerative disease has led to increased interest in alternative therapies. Cord blood plasma (CBP) derived from human umbilical cord blood (hUCB) may be a potential therapeutic. Benefits of CBP injection into rodent models of aging or ischaemic stroke have been demonstrated, though how benefits are elicited is still unclear. The present study evaluated various factors within the same samples of CBP and human adult blood plasma/sera (ABP/S). Also, autologous CBP effects vs. ABP/S or foetal bovine serum supplements on mononuclear cells from hUCB (MNC hUCB) in vitro were determined. Results showed significantly low concentrations of pro‐inflammatory cytokines (IL‐2, IL‐6, IFN‐γ, and TNF‐α) and elevated chemokine IL‐8 in CBP. Significantly higher levels of VEGF, G‐CSF, EGF and FGF‐basic growth factors were determined in CBP vs. ABP/S. Autologous CBP media supplements significantly increased MNC hUCB viability and decreased apoptotic cell activity. We are first to demonstrate the unique CBP composition of cytokines and growth factors within the same CBP samples derived from hUCB. Also, our novel finding that autologous CBP promoted MNC hUCB viability and reduced apoptotic cell death in vitro supports CBP's potential as a sole therapeutic or cell‐additive agent in developing therapies for various neurodegenerative diseases.

## 1 INTRODUCTION

Cord blood plasma (CBP) is commonly obtained from human umbilical cord blood (hUCB) during cell isolation and has mainly been considered a waste product. However, the trophic effect of CBP has been shown in replacing standard serum during the expansion of hUCB‐derived mesenchymal stem cells,^1^ human dental stem cells,^2^ hUCB‐derived T‐lymphocytes,^3^ or human endothelial colony‐forming cells^4^ in vitro. Moreover, the therapeutic potential of CBP administration into rats modelling acute ischaemic stroke was demonstrated by enhancement of neurogenesis and reduction of inflammation leading to significant post‐stroke functional recovery.^5^ Also, tissue inhibitor of metalloproteinases 2, a plasticity‐enhancing protein from CBP, has been found to promote restoration of hippocampal function and memory in aged 18 months old mice after CBP treatment.^6^ A recent study^7^ showed beneficial functional improvement in an Alzheimer's disease (AD) mouse model by injection of a specific fraction from cord blood serum compared to adult blood serum. Additionally, umbilical cord serum has being effectively employed for the treatment of corneal defects^8^, ^9^ and neurotrophic keratitis^10^ in humans.

In a relatively recent study,^11^ we showed the ability of CBP to modulate mitogen‐induced in vitro proliferation of mononuclear cells (MNC) isolated from the peripheral blood of amyotrophic lateral sclerosis (ALS) patients. Interestingly, three distinct cell responses to the mitogenic factor phytohemmagglutinin were noted, suggesting altered lymphocyte functionality in ALS patients. MNC responses were shown to be regulated by CBP treatment in vitro. Additionally, the apoptotic activity of MNCs isolated from ALS patients was significantly reduced by supplementing media with CBP. Thus, these study results have not only broadened the therapeutic application of CBP for ALS, but also further expanded its potential for treatment of other neurodegenerative disorders with immunological aspects.

It has been shown that CBP contains high amounts of various growth factors, such as vascular endothelial growth factor (VEGF), insulin‐like growth factor‐1 and transforming growth factor (TGF)‐β, that are required for cell maintenance during hematopoiesis.^3^, ^12^ Although CBP can exert a favourable effect on hematopoietic stem cells, whether CBP elicits therapeutic benefit as an additive to, or substitute for, cells must be determined before developing clinically relevant CBP‐based therapies for various neurodegenerative diseases.

The aim of this study was to characterize the composition of factors in CBP derived from hUCB, which might mediate therapeutic benefit. First, cytokine and growth factor profiles were analyzed in the same CBP samples. Second, the efficacy of autologous CBP on MNC hUCB viability in vitro was investigated. Finally, the effect of autologous CBP upon the apoptotic MNC hUCB response in vitro was examined. These study results provide a basis for further establishment of CBP as a potential self‐contained therapeutic or as a supportive diluent for MNC hUCB infusion in treatment of neurodegenerative diseases.

## 2 MATERIALS AND METHODS

### 2.1 Ethics statement

The human umbilical cord blood (hUCB) units were collected by Texas Cord Blood Bank (TCBB, GenCure, West San Antonio, TX, USA) and provided to Saneron CCEL Therapeutics, Inc. for research purposes. The cord blood units were obtained from full‐term pregnancies by vaginal delivery. The umbilical cord blood units were received within 48 hours of collection. Maternal blood samples, collected as the same time as the cord blood, were tested by TCBB for infectious disease markers of HIV, hepatitis B and C, syphilis, CMV and HTLV I&II, and test results were provided for validation of the cord blood units. Each cord blood unit in the study was negative for all infectious disease markers as determined in maternal blood. Human adult blood plasma or sera (ABP/S) was obtained from a commercially available source (Sigma‐Aldrich, St. Louis, MO, USA). Upon receipt of ABP/S, samples were aliquoted and stored at −20°C.

### 2.2 Human umbilical cord blood processing and plasma isolation

Human umbilical cord blood (hUCB) units (n = 20), with maternal blood samples negative for all tested infectious markers, were processed to obtain an autologous CBP fraction and mononuclear cell population (MNC hUCB, U‐CORD‐CELL™, Saneron CCEL Therapeutics, Inc., Tampa, FL, USA) as detailed below. Upon receipt, the cord blood units were diluted (1:1) with sterile phosphate buffered saline (PBS) without Mg^2+^ or Ca^2+^ (Sigma‐Aldrich, St. Louis, MO, USA). The cord blood was then fractionated using the density gradient solution Ficoll (Ficoll‐Paque Premium: 1.078 g/mL, Cat. No. 17544202; MilliporeSigma, St. Louis, MO, USA) in the Sepax 2 fully automated cell processing system (Biosafe America Inc., Houston, TX, USA). This allowed for the sterile collection of both CBP and MNC hUCB from each unit of cord blood. The CBP was further centrifuged at 3000 *g* for 10 minutes to remove any additional red blood cells. The CBP was then aliquoted and stored at −20°C. The MNC hUCB cell numbers and viability were determined using the Vi‐CELL Viability Analyzer (Beckman Coulter, Brea, CA, USA). MNC hUCB was then frozen at 5 × 10^7^ cells per vial using a proprietary cryopreservation media (Saneron CCEL Therapeutics, Inc.) and stored in liquid nitrogen.

### 2.3 Cytokine profile in human umbilical cord blood plasma

A human ultrasensitive cytokine 10‐plex panel (Invitrogen, Carlsbad, CA, USA; Cat. No. LHC6004) was used as previously described^13^ to determine the concentrations of cytokines within CBP (n = 20) and ABP/S (n = 6) in triplicate, following the manufacturer's protocol. All measurements were performed by an investigator blinded to the sample source. Granulocyte‐macrophage colony‐stimulating factor (GM‐CSF) and cytokine levels of interleukin (IL)‐1β, IL‐2, IL‐4, IL‐5, IL‐6, IL‐8, IL‐10, interferon‐gamma (IFN‐γ), tumour necrosis factor‐alpha (TNF‐α) and GM‐CSF were quantified using the Bio‐Rad Bio‐Plex^®^ Luminex 200 multiplex assay system (Bio‐Rad Laboratories Inc., Hercules, CA, USA). The Bio‐Rad Bio‐Plex^®^ 200 software (BioRad Laboratories Inc., Hercules CA, USA) was used to calculate the sample cytokine concentrations according to a standard curve and results were presented as picograms of analyte per milliliter (pg/mL).

### 2.4 Growth factor profile in human umbilical cord blood plasma

A human growth factor 4‐plex panel (Invitrogen; Cat No. LHC0007) was employed to determine various growth factor levels within CBP (n = 20) and ABP/S (n = 6) samples in triplicate, following the manufacturer's protocol. All measurements were performed by an investigator blinded to the source of the samples. Levels of VEGF, granulocyte colony‐stimulating factor (G‐CSF), epidermal growth factor (EGF) and fibroblast growth factor basic (FGF‐basic) were determined using the Bio‐Rad Bio‐Plex^®^ Luminex 200 multiplex assay (BioRad Laboratories Inc., Hercules CA, USA). The Bio‐Rad Bio‐Plex^®^ 200 software (BioRad Laboratories Inc., Hercules CA, USA) was used to calculate the sample growth factor concentrations accordingly to a standard curve and results were presented as pg/mL.

### 2.5 Viability of MNC hUCB cultured with autologous CBP

Cryopreserved MNC hUCB cells (n = 4 units) were quickly thawed at 37°C, washed with PBS, and centrifuged at 400 *g* for 5 minutes. Cell quantity and viability were determined using a haemocytometer. The cells were then re‐suspended with phenol‐free RPMI‐1640 media (Gibco, Dublin, Ireland; Cat. No. 11835030) and plated in a 24‐well cell culture plate at a density of 5 × 10^4^ cells/well. Pre‐designated wells were supplemented with 10% of autologous CBP, ABP/S, or foetal bovine serum (FBS) (Gibco, Dublin, Ireland; Cat No. 10438026) upon initial plating in duplicate. Cells were incubated at 37°C with 5% CO_2_ for 5 days. Media was changed at 24 hours and 3 days after cell plating. On day 5, cell viability was determined using the LIVE/DEAD viability/cytotoxicity kit (Molecular Probes, Cat No. R37601) accordingly to the manufacturer's instructions. Briefly, the culture media was replaced with 250 μL of fresh PBS in each well. In an equal volume to PBS, LIVE/DEAD working solution (250 μL) was added to each well and incubated at 37°C for 30 minutes. After incubation, confocal microscopy images (n = 3‐4/well, totalling n = 16‐20/supplement, mainly from the middle of the well) of cell fluorescence were obtained at 10x magnification for cell quantification using the Olympus FluoView 1000 confocal laser scanning microscope (Olympus Corporation of the Americas, Center Valley, PA, USA). Live cells were labelled with green fluorescence through the conversion of non‐fluorescent cell‐permanent calcein acetoxymethyl to intensely fluorescent calcein by ubiquitous intracellular esterase enzyme activity. Dead cells were identified using ethidium homodimer‐1, which enters cells through damaged membranes and produces a red fluorescence upon binding to nucleic acids. Cell counts of live (green) and dead (red) cells were determined using NIH ImageJ software (version 1.46).

### 2.6 Apoptotic activity of MNC hUCB cultured with autologous CBP

Cryopreserved MNC hUCB cells (n = 6 units) were quickly thawed at 37°C, washed with PBS, and centrifuged at 400 *g* for 5 minutes. Cell quantity and viability were determined using a haemocytometer. Cells were then re‐suspended with phenol‐free RPMI‐1640 media and plated in a 96‐well culture plate at a density of 2 × 10^4^ cells/well. Pre‐designated wells were supplemented with 10% of either autologous CBP, ABP/S, or FBS upon initial plating in duplicate. Cells were incubated at 37°C with 5% CO_2_ for 5 days. Media was changed at 24 hours and 3 days after cell plating. On day 5, the apoptotic activity of the cells was determined using the HT TiterTACS™ Assay kit (Trevigen, Bio‐Techne, Minneapolis, MN, USA; Cat No. 4822‐96‐K) accordingly to the manufacturer's instructions. Briefly, the cells were washed with 200 μL of sterile PBS, then quickly fixed using a 3.7% PBS buffered formaldehyde solution. The cells were washed once more with PBS and then permeabilized with Cytonin™ (50 μL/well). TACS‐Nuclease™ (50 μL/well) was then added to designated wells to determine total absorbance. The plate was incubated for 60 minutes at 37°C following a wash with PBS. The endogenous peroxidase activity was quenched with a 3% hydrogen peroxide solution. The wells were then washed once more with PBS and a 1x TdT labelling buffer reaction mix was added to the wells and incubated at 37°C for 60 minutes. To stop the labelling reaction, 1x TdT stop buffer was added to the well and incubated for 5 minutes, followed by a wash with PBS. The streptavidin‐HRP enzyme solution was then added to the wells and incubated for 10 minutes at RT. After an additional wash with PBS, the TACS‐Sapphire substrate solution was added and incubated for 30 minutes at RT with light protection. Stop solution of 0.2N HCl was added to each well and absorbance at 450 nm was measured using a spectrophotometer (SpectraMax Plus 384 microplate reader, Molecular Devices, LLC., San Jose, CA, USA). Results were calculated as the percentage of relative apoptotic absorbance values to maximum absorbance values determined for each culture condition. Cell morphology was observed using phase contrast images (n = 6/supplement) obtained at 20x using an Olympus IX70 inverted microscope (Olympus Corporation of the Americas, Center Valley, PA, USA).

### 2.7 Statistical analysis

Data was presented as mean ± SEM Statistical analysis was performed using GraphPad Prism Software version 5 (GraphPad Software, Inc.). The results for MNC hUCB viability and apoptotic activity were evaluated using a one‐way ANOVA with Tukey's Multiple Comparison post‐hoc test. The results for cytokine and growth factors in CBP were analyzed with a two‐tailed *t* test using same software. A value of *P* < 0.05 was considered significant.

## 3 RESULTS

### 3.1 Cord blood plasma cytokine profile

**Figure 1:**
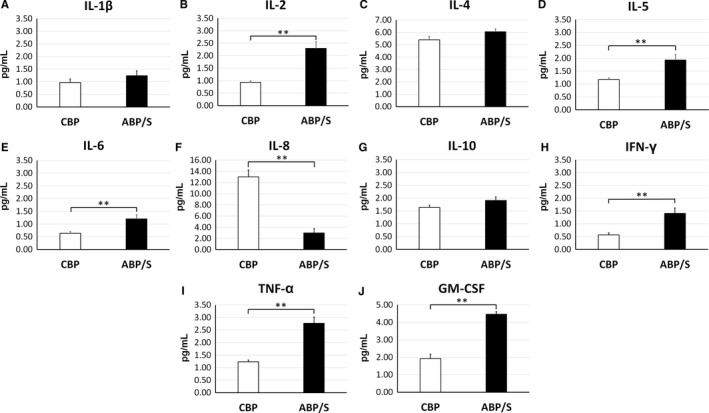
Cord blood plasma cytokine profile. The cytokine profiles of CBP (n = 20) and ABP/S (n = 6) were assayed using an ultrasensitive human cytokine panel in triplicate. Significantly lower concentrations of the pro‐inflammatory cytokines (B) IL‐2, (E) IL‐6, (H) IFN‐γ and (I) TNF‐α were detected in CBP vs. ABP/S. Levels of immunomodulatory (D) IL‐5 cytokine and (J) GM‐CSF were significantly low in CBP. A significant increase in (F) IL‐8 was also determined in CBP. There were no significant differences between CBP and ABP/S for (A) IL‐1β, (C) IL‐4 and (G) IL‐10. **P < 0.01

Samples of CBP and ABP/S were assayed to determine cytokine profiles using an ultrasensitive human cytokine 10‐plex panel. Results showed significantly (*P* < 0.01) lower concentrations of the pro‐inflammatory cytokines IL‐2, IFN‐γ and TNF‐α in CBP compared to ABP/S (Figure 1B,H,I). Additionally, levels of immunomodulatory IL‐5 (Figure 1D) and multifunctional IL‐6 (Figure 1E) cytokines were also significantly (*P* < 0.01) lower in CBP vs. ABP/S. Significantly (*P* < 0.01) elevated concentrations of the chemokine IL‐8 were determined in CBP in comparison in ABP/S (Figure 1F). Interestingly, levels of the pro‐inflammatory immune cell maturating factor, GM‐CSF, were significantly (*P* < 0.01) lower in CBP than in ABP/S (Figure 1J). Although the levels of IL‐1β, IL‐4 and IL‐10 were slightly reduced in CBP compared to ABP/S, these reductions were not statistically significant (*P* > 0.05) (Figure 1A,C,G). While anti‐inflammatory IL‐4 and IL‐10 cytokine concentrations were not significantly different between CBP and ABP/S, it is important to note that most of the pro‐inflammatory cytokines within CBP were present at lower concentrations than their anti‐inflammatory counterparts. Concentrations of cytokines in CBP and ABP/S are provided in Table 1A.

**Table 1:** Cytokine and growth factor profiles in cord blood plasma and adult blood plasma/serum

A. Cytokine profile (pg/mL)			B. Growth factor profile (pg/mL)		
Cytokine	CBP	ABP/S	Growth factor	CBP	ABP/S
IL‐1β	0.97 ± 0.14	1.24 ± 0.19	VEGF	7.23 ± 0.28**	2.94 ± 0.11
IL‐2	0.93 ± 0.05**	2.29 ± 0.27	G‐CSF	59.89 ± 2.26*	46.22 ± 0.52
IL‐4	5.40 ± 0.28	6.05 ± 0.23	EGF	11.00 ± 0.41**	4.64 ± 0.13
IL‐5	1.17 ± 0.07**	1.93 ± 0.22	FGF Basic	6.07 ± 0.18**	2.35 ± 0.12
IL‐6	0.64 ± 0.07**	1.20 ± 0.17			
IL‐8	13.02 ± 1.22**	2.98 ± 0.79			
IL‐10	1.64 ± 0.08	1.91 ± 0.14			
IFN‐γ	0.57 ± 0.08**	1.41 ± 0.21			
TNF‐α	1.23 ± 0.07**	2.77 ± 0.25			
GM‐CSF	1.91 ± 0.26**	4.46 ± 0.15			

Levels of cytokines and growth factors presented as mean ± SEM.

CBP: Cord Blood Plasma; ABP/S: Adult Blood Plasma/Serum; Interleukin (IL): 1β, 2, 4, 5, 6, 8, and 10; IFN‐γ: Interferon‐gamma; TNF‐α: Tumour necrosis factor‐alpha; GM‐CSF: Granulocyte‐macrophage colony stimulating factor; VEGF: Vascular endothelial growth factor; G‐CSF: Granulocyte‐colony stimulating factor, EGF: Epithelial growth factor; FGF Basic: Fibroblast growth factor basic.

Significance of CBP vs. ABP/S denoted by: *P <* *0.05; **P <* *0.01.

### 3.2 Cord blood plasma growth factor profile

**Figure 2:**
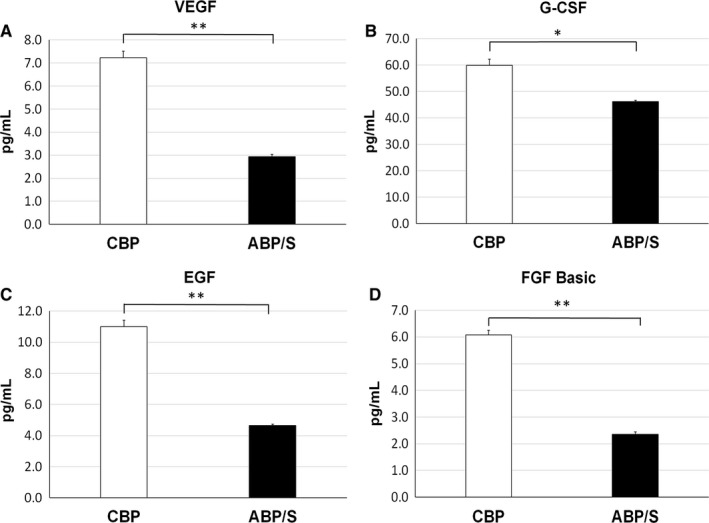
Cord blood plasma growth factor profile. The levels of the growth factors were analyzed in CBP (n = 20) and ABP/S (n = 6) using a human growth factor multiplex assay in triplicate. Significantly higher concentrations of (A) VEGF, (B) G‐CSF, (C) EGF and (D) FGF basic growth factors were detected in CBP vs. ABP/S. *P < 0.05, **P < 0.01

The levels of several common growth factors were measured in CBP and ABP/S using a human growth factor four‐plex assay. The concentrations of VEGF were significantly (*P* < 0.01) higher in CBP, more than two‐fold, vs. ABP/S (Figure 2A). The concentrations of G‐CSF, a bone marrow stem cell stimulating growth factor, were also significantly (*P* < 0.05) higher in CBP compared to ABP/S (Figure 2B). Also, the cell proliferating growth factors: epidermal growth factor (EGF) and fibroblast growth factor basic (FGF‐basic) were significantly (*P* < 0.01) elevated in CBP (Figure 2C,D; respectively). Of note, the levels of EGF and FGF‐basic factors were about 2.5‐fold higher in CBP vs. ABP/S. Levels of growth factors in CBP and ABP/S are indicated in Table 1B.

### 3.3 Viability of MNC hUCB cultured with autologous CBP

**Figure 3:**
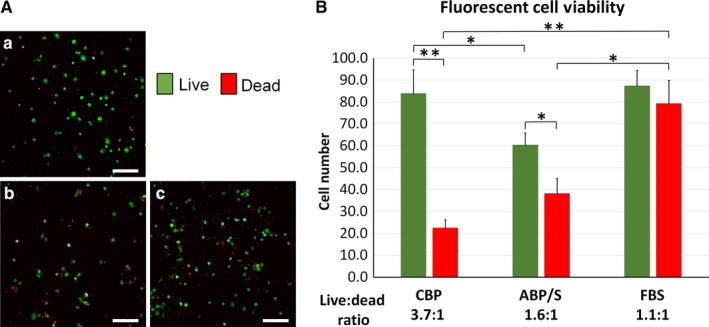
Viability of MNC hUCB in vitro. MNC hUCB (n = 4 units) was cultured for 5 d in media supplemented with either autologous CBP, ABP/S, or FBS in duplicate. The cells were stained using the LIVE/DEAD Viability/Cytotoxicity assay to identify the viable (green fluorescence) and non‐viable cytotoxic (red fluorescence) cell populations from images totalling n = 16‐20/supplemental condition. A, Confocal microscopy images demonstrated numerous viable (green) MNC hUCB cultured with (Aa) CBP and (Ac) FBS supplements. Fewer viable cells were detected in culture supplemented with (Ab) ABP/S. Scale bar in Aa‐Ac is 100 μm. (B) MNCs cultured with autologous CBP supplement showed significantly greater cell survival vs. ABP/S. Also, media supplemented with CBP showed significantly reduced numbers of dead (red) MNC hUCB compared to FBS. Cells supplemented in media with CBP had a greater live (green)/dead (red) cell ratio compared to cultures that received ABP/S or FBS.*P < 0.05, **P < 0.01

Cryopreserved MNC hUCB was incubated with RPMI‐1640 media supplemented with autologous CBP, ABP/S, or FBS for 5 days. After 5 days in vitro, the cells were stained using the LIVE/DEAD Viability/Cytotoxicity assay to identify the viable (green fluorescence) and non‐viable cytotoxic cell populations (red fluorescence). Numerous viable MNC hUCB were observed in cultures with CBP (Figure 3Aa) and FBS (Figure 3Ac) supplements. Fewer viable cells were seen with ABP/S (Figure 3Ab) added into media. Live cell counts of MNC hUCB supplemented with autologous CBP were significantly (83.83 ± 10.86 cell number, *P* < 0.05) higher vs. cultured cells supplemented with ABP/S (60.35 ± 5.50 cell number, Figure 3B). However, numbers of viable cells cultured with CBP (83.83 ± 10.86 cell number) and FBS (87.33 ± 7.17 cell number) were similar (Figure 3B). Importantly, media supplemented with CBP showed significantly (*P* < 0.01) reduced numbers of dead MNC hUCB (22.50 ± 3.67 cell number) compared to FBS (79.33 ± 10.48 cell number). Yet, MNC hUCB cultured with FBS demonstrated a significant (*P* < 0.05) increase of dead cells vs. cultured cells supplemented with ABP/S (38.15 ± 6.90 cell number, Figure 3B). Additionally, cells supplemented in media with CBP had a greater ratio of live to dead cells (3.7:1) compared to cultures that received ABP/S (1.6:1) or FBS (1.1:1).

### 3.4 Apoptotic activity of MNC hUCB cultured with autologous CBP

**Figure 4:**
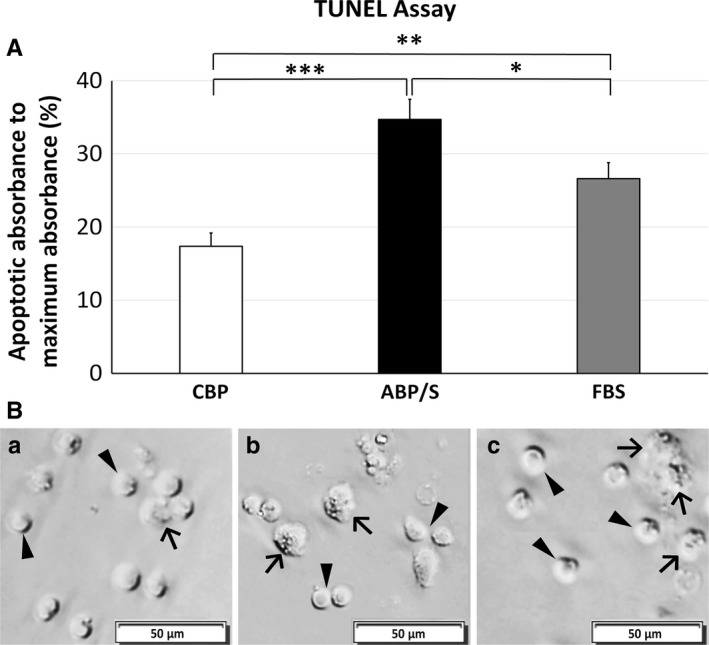
Apoptotic activity of MNC hUCB in vitro. MNC hUCB (n = 6 units) was cultured for 5 d in media supplemented with either autologous CBP, ABP/S, or FBS in duplicate. Apoptosis was detected by TUNEL assay. A, MNCs cultured in autologous CBP showed a significantly lower percentage of apoptotic absorbance vs. cultures supplemented with ABP/S or FBS. Cells incubated with FBS also exhibited significantly lower absorbance of apoptotic activity compared to ABP/S. *P < 0.05, **P < 0.01, ***P < 0.001. B, Phase contrast images of MNC hUCB in vitro demonstrated a few cells with abnormal morphology displaying dislocated nuclei in cultures supplemented with (Ba) CBP compared to numerous morphologically damaged cells cultured with (Bb) ABP/S or (Bc) FBS, supporting apoptotic cell counts. Arrowheads indicate healthy cells with normal morphology. Arrows indicate cells with abnormal morphology. Scale bar in Ba‐Bc is 50 μm

Apoptotic activity of cultured MNC hUCB in media supplemented with autologous CBP, ABP/S, or FBS was analyzed on day 5 in vitro using a colormetric TUNEL assay. The percentage of apoptotic cells cultured with CBP was significantly lower (17.39 ± 1.70%) compared to cultures supplemented with ABP/S (34.72 ± 2.61%, *P* < 0.001) or FBS (26.62 ± 2.08%, *P* < 0.01) (Figure 4A). Interestingly, MNC hUCB cultured in media containing FBS showed significantly (*P* < 0.05) lower apoptotic activity vs. cultured cells with ABP/S. Phase contrast microscopic images of MNC hUCB in vitro demonstrated a few cells with abnormal morphology displaying dislocated nuclei in cultures supplemented with CBP (Figure 4Ba) compared to numerous morphologically damaged cells cultured with ABP/S (Figure 4Bb) or FBS (Figure 4Bc), supporting apoptotic cell counts.

## 4 DISCUSSION

In the present study, various factors in CBP derived from hUCB and the effect of CBP on mononuclear cells isolated from hUCB (MNC hUCB) in vitro were evaluated in the context of establishing CBP as a potential therapeutic agent. Cytokine and growth factor profiles were examined within the same samples of CBP and human adult blood plasma/sera (ABP/S). The effect of autologous CBP on MNC hUCB in vitro was determined and compared to ABP/S and standard FBS media supplements. The major study findings were that CBP demonstrated: (a) significantly “low” concentrations of the pro‐inflammatory cytokines IL‐2, IL‐6, IFN‐γ, and TNF‐α; (b) significantly “low” concentrations of immunomodulatory IL‐5 cytokine and GM‐CSF; (c) significantly “elevated” level of the chemokine IL‐8; (d) significantly high concentrations of VEGF, G‐CSF, EGF and FGF‐basic growth factors; (e) significantly “increased” viability of MNC hUCB in vitro with autologous CBP media supplement; and (f) significantly “decreased” apoptotic MNC hUCB in vitro with autologous CBP media supplement. To our knowledge, we are the first to demonstrate the unique CBP composition of cytokines and growth factors within the same samples, providing evidence of the unique protein content in CBP. Also, our novel finding is that autologous CBP promoted MNC hUCB viability and reduced apoptotic cell death in vitro, supporting the notion that CBP has potential as a sole therapeutic or cell‐additive agent in developing clinically relevant CBP‐based therapies for various neurodegenerative diseases.

In development of alternative approaches in treatment for age‐related diseases, proteins from “young” blood have been intensely investigated. Studies of parabiosis, with shared blood circulatory systems between old (16‐20 months of age) and young (2‐3 months of age) mice, have shown significantly improved cognition and physical function in both aged wild‐type mice^14^ and a mouse model of Alzheimer's disease (AD).^15^ Middeldorp et al^15^ demonstrated that parabiosis of young wild‐type mice with AD mice for 5 weeks effectively improved learning and memory while also reducing inflammation in AD mice. Additionally, the authors noted increased synaptic activity in the hippocampus of AD mice. Based on these study results, clinical trial (NCT02256306) investigated the safety of 4‐weekly infusions of young blood plasma from donors aged between 18 and 30 years of age into patients with AD. Although no serious adverse reactions occurred, the study found no significant effect on patient cognition but did show significant improvements in daily living skills. Although results of using young blood are promising, it is still unclear which constituents of “young” blood are providing beneficial effects. Potentially, paracrine actions are involved in positive outcomes for treatment of an age‐related disease such as AD. Also, hormonal status of donors should be investigated due to the wide age range (18‐30 years) of donors. Alternatively, plasma derived from hUCB could be a more beneficial therapeutic due to its unique and uniform molecular composition.

It has been shown that in addition to a high concentration of growth factors (reviewed^16^), human CBP also contains a great amount of soluble proliferative and immunomodulatory factors such as (TGF)‐β, G‐CSF, GM‐CSF, monocyte chemoattractant protein (MCP)‐1, IL‐6, and IL‐8.^17^ Also, IL‐16 cytokine, a modulator of T cell activation, has been detected in CBP^18^ and potentially presents a physiological mechanism for foetal‐maternal tolerance. Due to CBP's specific molecular composition, numerous studies^1^, ^2^, ^3^, ^4^ showed beneficial effect of CBP in replacement of standard FBS for various cell expansions in vitro, which may be essential to achieve appropriate cell numbers for clinical use.

In our study, cytokine and growth factor levels were analyzed in the same CBP samples for a better understanding of CBP molecular composition prior to proposing CBP as a therapeutic agent. We showed low concentrations of pro‐inflammatory IL‐2, IL‐6, IFN‐γ and TNF‐α cytokines in CBP, presumably secreted by various cells in hUCB, which, likely signify the immune immaturity of these cell populations. Additionally, our study findings demonstrated a significantly low concentration of immunomodulatory cytokine IL‐5 in CBP vs. ABP/S, supporting previous study results.^19^ This cytokine, mainly produced by Th2 helper lymphocytes and mast cells, promotes growth/differentiation of B cells and granulocytes upon immunological and/or antigenic priming in development of the adaptive immune response. However, increased concentrations of IL‐5, IL‐2 and transcription factor GATA‐4 determined in CBP may result in abnormal patterns of foetal immune system development and induce risk of allergic disease.^20^ Also, it has been shown that antioxidant capacity, evaluated by carbonyl levels in CBP, was significantly higher in patients delivering neonates by caesarean vs. vaginal route, suggesting that the delivery method impacts oxidative stress.^21^ In our study, the low concentration of GM‐CSF found in CBP together with the low concentrations of pro‐inflammatory cytokines provide further evidence of anti‐inflammatory hUCB content. Thus, low levels of pro‐inflammatory and immunomodulatory cytokines in CBP provide a favourable microenvironment for cellular content in hUCB. It has been shown that transplantation of MNC derived from hUCB even from unrelated donors into patients with haematologic malignancies causes a low incidence of graft‐versus‐host disease compared to bone marrow or peripheral blood cell administration.^22^, ^23^

Our study results also demonstrated similar amounts of anti‐inflammatory IL‐4 and IL‐10 cytokines in CBP and ABP/S, However, it is important to note that these anti‐inflammatory cytokines were present at a greater concentration than the pro‐inflammatory constituents of CBP, suggesting a favourable cytokine composition towards developing CBP as potential therapeutic agent. Since IL‐10 is an important cytokine for downregulation of Th1 inflammatory cytokines and MHC class II antigens, a decrease of this cytokine is mainly associated with altered cell‐mediated immunosuppression and induction of complications during pregnancy.^24^ In contrast, increased cord blood IL‐10 was determined in preterm infants compared to full‐term newborns.^25^, ^26^ In our study, hUCB units were used from healthy infants delivered naturally, so IL‐10 levels determined in CBP vs. ABP/S likely reflect steady immune/inflammatory humoral status in hUCB.

Amongst our additional important study findings were significant elevations of VEGF, G‐CSF, EGF and FGF‐basic growth factors in CBP vs. ABP/S. Both EGF and FGF‐basic promote stem cell renewal and inhibit cell senescence^27^ and elevated levels of EGF largely correlate to gestational age and birth weight of the developing foetus.^28^, ^29^ Thus, the increased levels of EGF and FGF‐basic in CBP determined in our study may indicate normal foetal development. Also, increased G‐CSF, a bone marrow stem cell mobilizing factor, in CBP potentially reflects intensive production of bone marrow derived stem cells in the foetus. The combination of this growth factor with MNC hUCB for the treatment of myeloid malignancies in human adults after radiation promoted cell engraftment in bone marrow replacement therapies.^30^, ^31^ Also, co‐administration of G‐CSF with MNC hUCB into an animal model of traumatic brain injury results demonstrated reduction of neuroinflammation and promotion of stem cells into the injured side of the brain.^32^

Of note, significantly elevated levels of the chemokine IL‐8 and VEGF were determined in CBP vs. ABP/S in our study. While IL‐8 is primarily known as a pro‐inflammatory mediator, it also recognized as a promoter of angiogenic activity as demonstrated by endothelial cell survival, proliferation and migration in vitro.^33^, ^34^ Interestingly, the concentration of the angiogenic VEGF growth factor was also significantly higher in CBP vs. ABP/S. It is possible that the elevated level of VEGF is a result of the high concentration of IL‐8, which promotes increased expression of VEGF by endothelial cells.^35^, ^36^ A recently published study^37^ demonstrated that microRNA‐containing exosomes derived from maternal and umbilical cord serum dramatically promote human umbilical vein endothelial cell proliferation, migration, and tube formation in vitro, highlighting the important role of exosomes in the regulation of angiogenesis during gestation. Exclusively, VEGF has been studied for potential therapeutic efficacy in animal models of ALS^38^, ^39^ and its use in clinical settings has been discussed (reviewed^40^). Nevertheless, CBP containing high levels of IL‐8 and VEGF might be a beneficial treatment for repair of the damaged blood–brain barrier and/or blood–spinal cord barrier in patients with ALS,^41^, ^42^, ^43^, ^44^ AD,^45^ Parkinson's disease^46^ and multiple sclerosis.^47^

Finally, our in vitro studies showed significantly increased viability of MNC hUCB when autologous CBP was added to culture media. Also, apoptotic activity of MNC hUCB in vitro, determined by TUNEL, was also decreased after autologous CBP exposure compared to cultures supplemented with ABP/S or FBS. Supporting this novel finding, our previous study has demonstrated reduced activities of other pro‐apoptotic factors, such as caspase 3/7, from ALS patient‐derived MNC's cultured in media supplemented with CBP.^11^

In this context, numerous studies have shown neuroprotective effects of MNC hUCB administered into animal models of ALS,^48^, ^49^, ^50^, ^51^ AD,^52^, ^53^, ^54^ Parkinson's disease,^55^ ischaemic stroke^56^, ^57^ and traumatic brain injury.^58^, ^59^ However, insignificant numbers of MNC hUCB were detected in the CNS of these animal models after intravenous cell administration. This scarcity is likely due to a low rate of cell survival, since cell preparation and injection involve dilution with a basic buffer solution. Substitution of this diluent with autologous CBP might present a more supportive microenvironment for cell survival and increase therapeutic efficacy of administered MNC hUCB. Especially, complementing MNC hUCB with autologous CBP may foster injected cell survival. Our in vitro study results on cell viability and apoptotic activity support this suggestion. Also, repeated administrations of MNC hUCB cells with autologous CBP may prove even more advantageous. Alternatively, injection of non‐autologous CBP alone might be efficacious for treatment of various neurodegenerative diseases and/or aging population per se. Beneficial effects have been observed from intravenous administration of CBP into rats modelling acute ischaemic stroke^5^ or into an animal model of ageing.^6^ In these studies, multiple injections of CBP were performed and this therapeutic approach needs to be considered. In agreement with this approach, repeated deliveries of CBP could provide ongoing trophic support for damaged cells and/or tissues. Our study showed that CBP is a potential therapeutic due to its unique composition. We are planning in the near future to determine the effect of CBP alone and in combination with MNC hUCB for treatment of ALS using a symptomatic animal model of disease for a translational perspective.

In conclusion, our study results demonstrate uniquely protein content in the same CBP samples composed of cytokines and growth factors. The novel in vitro finding of autologous CBP with MNC hUCB demonstrated the trophic capacity of this combination through promotion of cell viability and reduction of apoptotic death. These findings further support the potential of CBP as an independent therapeutic or cell‐additive agent in clinical applications for various neurodegenerative diseases.

## CONFLICT OF INTEREST

PRS is a co‐founder and SGD is a consultant for Saneron CCEL Therapeutics, Inc. JE is the Director of Research and Development for Saneron CCEL Therapeutics, Inc. PRS and SGD have patents for the application of hUCB as a cell therapy for several disorders.

## AUTHOR CONTRIBUTIONS

JE and SGD designed the studies and wrote the manuscript. JE performed all assays and data analysis. PRS participated in study design and discussion of results. All authors reviewed the manuscript.
